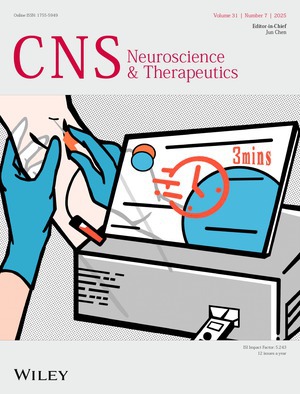# Front Cover

**DOI:** 10.1111/cns.70539

**Published:** 2025-07-24

## Abstract

The cover image is based on the article *Real‐Time Monitoring of the Antiseizure Drug Valproic Acid Using a Novel Point‐Of‐Care Mass Spectrometry* by Xinqi Fang et al., https://doi.org/10.1111/cns.70499.